# Surgery in the Hospitals after the Battle of Buena Vista

**Published:** 1847-12

**Authors:** W. B. Herrick

**Affiliations:** Professor of Anatomy in Rush Medical College, and late Surgeon to 1st Regiment Illinois Volunteers


### ARTICLE VII.

*Surgery in the Hospitals after the battle of Buena Vista.* By W. B. HERRICK, M. D., Professor of Anatomy in Rush Medical College, and late Surgeon to 1st Regiment Illinois Volunteers.

On the day of the battle, the wounded, after receiving such attention from the surgeons, as could, at the time and under the circumstances, be given to them, were removed from the field, some to Saltillo,* others to the Ranchea de Buena Vista.

When, on the evening of that day, we arrived at the last named place, we found those of the wounded still living, the dying and the dead, crowded together indiscriminately, and presenting a melancholy picture of suffering

* In the third line of our communication in the preceding number of this Journal, headed "Surgery at Buena Vista," for "Agua Nueva, ten miles from San Luis Potosi," read Agua Nueva, ten miles from Saltillo, on the road to San Luis Potosi.

and distress, not easily described, and never to be forgotten.

As soon as circumstances would permit, such as were still living, were selected from their less fortunate companions, placed in wagons in charge of the writer, and conveyed, late at night, to the large cathedral in town, which had already been converted into a temporary hospital.

The time, both day and night, for the forty-eight hours after, was spent by the surgeons in attending to such wounds as had been overlooked on the field, in renewing the dressings of those requiring attention, and in performing amputations and such other operations, as, upon a re-examination of the cases, seemed to be required.

During the third, and a part of the fourth days subsequent to the battle, the wounded were removed from the large but crowded church, to more convenient buildings, each capable of containing from fifty to one hundred patients.

In making this more permanent arrangement, those of the volunteers belonging to the same State, were, as far as was practicable, collected together in the same Hospital, and placed in charge of some one of their respective surgeons.

The wounded of the first and second Illinois regiments, consisting in all of nearly one hundred, in the proportion of about ten officers in their private quarters in different parts of the town, to eighty or ninety privates, collected together in one of the best and most commodious of the above named hospitals, came, in accordance with the plan adopted, naturally under our charge.

Myself, and so far as I know, all other surgeons in charge of hospitals, on making the proper requisitions, were provided with assistants, attendants, hospital stores, provisions, &c., promptly, and to an extent creditable both to the officers in charge, and to our country.

With regard to the subsequent termination and treatment of the cases under the charge of myself and others, it may be stated that most of the simple flesh wounds healed rapidly and kindly under the use of dressings of lint, changed once in twenty-four or forty-eight hours, with an occasional aperient, and a diet adapted to the case. In some few instances the tendency to inflammatory action was so great as to require general antiphlogistic treatment, and the use of emolients, anodynes, and other soothing local applications

The presence of foreign substances in the simplest wounds, frequently caused protracted suppuration, and the formation of abscesses, and thus retarded their cure, in many instances, for a long time.

Stimulants and tonics, such as brandy, wine, iron, and acids, were used freely by myself and others, and with maked benefit in all cases of debility consequent upon profnse suppuration. (A friend who has observed the beneficial effects of such treatment in one or two cases of the kind, suggests the propriety of using astringents, in the form of lotions or injections, to moderate the profuse and debilitating discharge from extensive suppurating surfaces.)

The gun shot wounds, in which bones were injured, presented all the complications of compound comminuted fractures, and proved to be the most troublesome and difficult cases to treat, The practice usually adopted by us, was to search carefully for and extract all detached pieces of bone, cleanse the wounds, bandage the limb firmly to a point high above the injury, and then to apply splints to prevent motion, but so as, if possible, not to interfere with the renewal of dressings, of the exit of pus. Thick paste board splints, shaped properly, perforated opposite to the external wounds, moistened and adapted accurately to the limb thus injured, afforded, when dry, a most perfect and firm support, and were found, therefore, to answer better than any others, the indications in such cases.

Shortening was, in a few instances, the necessary consequence of loss of portions of bone, but these were exceptions to the general rule, for in most cases of fracture, union took place without loss of motion or deformity.

In our last communication we remarked, that in a majority of instances, primary amputations were followed by favorable, and secondary, by unfavorable results. The principal reasons why this was the case are obviously the following: The most of the amputations upon the field, the only ones truly primary, were performed upon individuals in good health, and under excitement, and, therefore, not in a condition to suffer from debility or nervous depression. The subjects of the subsequent operations, on the other hand, had become depressed both physically and mentally by profuse suppuration, pain, and want of rest, and were, therefore, cases in which unfavorable results are most generally expected.

Most of the amputations in the hospitals were performed after consultations, and were found to be indicated, in most cases, not by the severity of the injury, so far as could be determined by the first examinations, but by the appearance of general symptoms of an unfavorable character, in many instances, such as are consequent upon the absorption of pus.

It is a remarkable fact, that upon making examinations in the only two cases which proved fatal after the first week under my care, one after amputation, balls were found lodged in cavities filled with pus, one at the upper, and the other at the lower extremity of the tibia.

In our opinion, the fatal termination of these cases, as indicated both by the symptoms and the examinations, was in consequence of the absorption of pus, for it must be admitted that pus globules, whether too large or not to be absorbed by uninjured vessels, might readily pass into the large venus sinuses, such as are found every where in large bones, when broken into as in the cases above cited.

In conclusion, I will state that the enemy had proved themselves to be mere barbarians, by murdering all of our wounded they had met upon the field, thus leaving alive but a small number of those most severely injured, consequently, the proportion of deaths to the number treated was comparatively small, not averaging, as we believe, after the first day or two, more than five or ten in a hundred.

In the hospitals which had been provided for the wounded Mexicans, and placed in charge of their own surgeons, a proportion of the cases, amounting to at least one half of the whole number, terminated fatally. This mortality among their wounded, resulted partly, doubtless, from the nature of their wounds, many of which had been produced by the balls, grape shot and shells from our large guns, but principally, as the effects of comparatively slight wounds upon naturally bad constitutions, rendered still worse by a diet composed of saccharine, fatty, and other carbonaceous substances, and a life of idleness and dissipation, such as they had been living during the six months previous, at San Luis Potosi.

In proof of this, it may be stated, that with them nearly all the cases in which amputations were performed, whether early or late, terminated fatally, many after troublesome hemorrhage from numerous small vessels, both during and after operations, and the entire absence of a tendency to a dhesion or healthy suppuration.

